# The effectiveness of a contingent financial incentive to improve trial follow up; a randomised study within a trial (SWAT)

**DOI:** 10.12688/f1000research.21059.2

**Published:** 2020-07-17

**Authors:** Catherine Arundel, Elizabeth Coleman, Caroline Fairhurst, Emily Peckham, Della Bailey, Simon Gilbody

**Affiliations:** 1 Department of Health Sciences, University of York, York, YO10 5DD, UK

**Keywords:** SWAT, retention, randomized controlled trial

## Abstract

**Objectives**

To evaluate the effectiveness of a contingent financial incentive (£10 note in addition to a routinely provided £10 voucher) versus no contingent financial incentive, on improving the retention rate in a randomised controlled trial (RCT).

**Methods**

A two arm ‘Study within a Trial’ (SWAT) embedded within a host RCT (SCIMITAR+). Participants were randomised to the SWAT using a 2:1 (intervention:control) allocation ratio. The primary outcome measure was the proportion of participants completing a CO breath measurement at the first SCIMITAR+ follow up time point (6 months). Secondary outcomes were withdrawing from follow-up after contact and time from assessment due date to completion.  Analyses were conducted using logistic or Cox Proportional Hazards regression as appropriate.

**Results**

A total of 434 participants were randomised into this SWAT. Completion of the CO breath measurement at 6 months was 88.5% (n=247) in the intervention arm of the SWAT and 85.4% (n=123) in the control arm (Difference 3.1%, OR 1.29, 95% CI 0.71-2.33, p=0.41). There was also no evidence of a difference in the proportion of participants withdrawing from follow-up after contact (intervention n=7 (2.5%), control n=5 (3.5%); OR 0.76, 95% CI 0.23-2.44, p=0.64), nor in terms of proximity of 6-month visit completion to due date (HR 1.07, 95% CI 0.86-1.33, p=0.55).

**Conclusion**

It is unclear if contingent financial incentives increased rates of face-to-face follow-up completion within the SCIMITAR+ trial population. However, the sample size of this SWAT was constrained by the size of the host trial and power was limited. This SWAT adds to the body of evidence for initiatives to increase response rates in trials.

## Introduction

Attrition is a major problem for randomised controlled trials (RCTs) with 25% experiencing more than 10% attrition ^1^.

Bower *et al.* (2014) ^2^ identified financial incentives as an effective retention strategy (RR 1.18; 95% CI 1.09 to 1.28), and effectiveness was increased if this incentive was provided on receipt of a completed questionnaire (RR 1.25; 95% CI 1.14 to 1.38). Bailey *et al.* (2013) ^3^ identified that varying the incentive level (£20 compared to £10) increased response to postal questionnaires by up to 10%.

Contingent incentives have shown to be effective in increasing questionnaire response rate, but have not been tested in relation to face-to face-visits. This SWAT evaluated the effectiveness of a contingent financial incentive - £10 cash in addition to a routinely provided £10 voucher - versus no contingent financial incentive, on improving the retention rate in the SCIMITAR+ trial.

## Methods

### Design

This SWAT was embedded within the SCIMITAR+ RCT which evaluated the effectiveness of a bespoke, individually-tailored, smoking cessation programme, compared to usual care, for adult smokers with severe mental ill health conditions ^4^. The SCIMITAR+ Trial was registered prospectively: ISRCTN72955454

This paper refers to the methods and results of the SWAT only.

### Participants

The SWAT ^5^ was conducted in 21 NHS Trusts and 16 primary care settings and was implemented after the start of SCIMITAR+ follow-up. Participants were eligible for this SWAT if they reached the SCIMITAR+ 6-month follow-up on or after 31 ^st^ September 2016.

### Intervention

When participants in the SWAT intervention group were contacted by the research team to arrange their follow-up appointment, they were advised of the potential of receiving £10 cash contingent on providing a carbon monoxide (CO) breath measure as part of their 6-month face-to-face study appointment, in addition to the £10 gift voucher routinely provided to all participants. Participants in both groups received all other pre-planned retention strategies within SCIMITAR+.

### Outcomes

The primary outcome for the SWAT was the proportion of participants completing a CO breath measurement at the SCIMITAR+ 6 month follow-up time-point. Secondary outcome measures were: i) the proximity of visit completion to visit due date; ii) the proportion of participants withdrawing from follow-up in the two months *after* initial contact was made to arrange the 6-month visit.

### Sample size

The sample size was determined by the number of participants followed-up at 6 months in SCIMITAR+ from the point at which this SWAT was embedded.

### Randomisation

Simple randomisation using random numbers was carried out by an independent statistician at the York Trials Unit using Stata v13 ^6^. All potentially participants eligible for inclusion, at the time of randomisation, were allocated with a 2:1 allocation ratio (intervention:control) due to the anticipated effectiveness of financial incentives increasing questionnaire response rates.

### Blinding

It was not possible to blind research staff to the participant’s allocation. Participants were not informed about the SWAT so were blind to the study hypothesis.

### Approvals

The SWAT was approved by the Research Ethics Committee Yorkshire and Humber – Leeds East (15/YH/0051). As the SWAT was deemed to be low risk, and to avoid disappointment for participants who did not receive the additional incentive, informed consent was not obtained for participation in this SWAT.

### Statistical analysis

Analyses were conducted using Stata v15 ^7^ on an intention to treat basis using two-sided statistical tests at the 5% significance level, adjusting for host trial allocation.

The proportion of participants who provided a 6-month CO breath measure was analysed using logistic regression. The odds ratio (OR), 95% confidence interval (CI) and p-value are presented.

The 6-month appointment due date was 183 days after randomisation. Participants who withdrew a month either side of the 6-month appointment due date were classed as withdrawn. The proportion of participants withdrawing from SCIMITAR+ in the two months after contact were analysed in the same way as the primary outcome.

A Cox Proportional Hazard model compared the proximity of the visit completion to visit due date (time in days). Participants who completed their visit before or on the due date had their time-to-visit set to 0.1.

## Results

**Figure 1.**
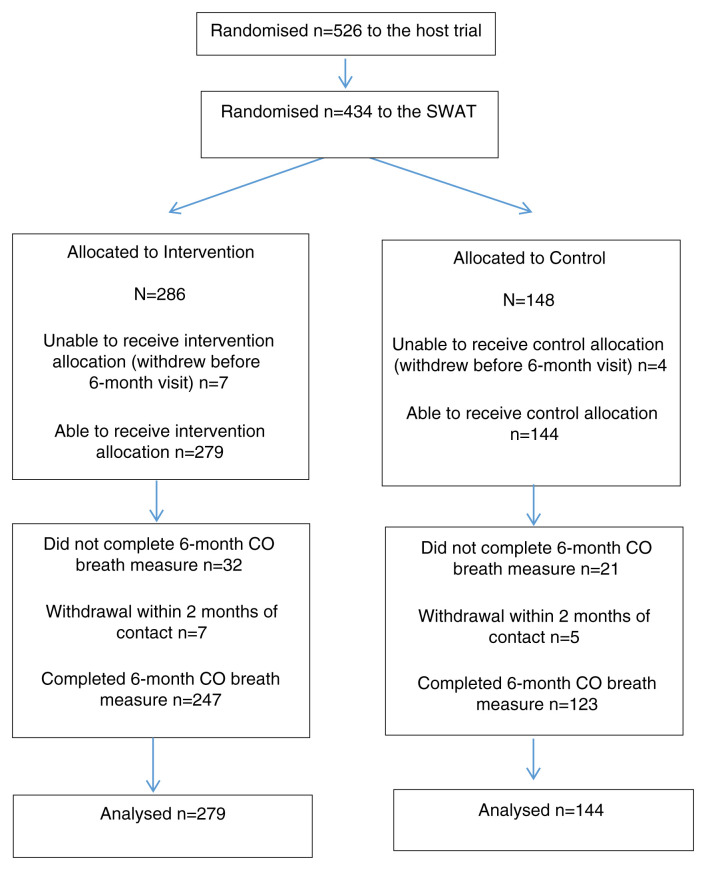
Study flow diagram.

In total, 434 participants were randomised into this SWAT (n=286, 65.9% intervention group; n=148, 34.1% control group). Due to randomisation occurring at a single time point, eleven participants were excluded from analysis as they withdrew from SCIMITAR+ following randomisation but prior to being contacted for their 6-month visit. There were 423 eligible participants (intervention group n=279, 66.0%; control group n=144, 34.0%) ( Figure 1).

Overall, 87.5% (n=370) of participants completed the CO breath measurement at 6 months and it was unclear if there was a difference between intervention (88.5%, n=247) and control groups (85.4%, n=123) (3.1% difference, OR 1.29, 95% CI 0.71-2.33, p=0.41). It was unclear if there was any difference in withdrawals between trials arms (intervention n=7, 2.8%; control n=5, 3.5%; OR 0.76, 95% CI 0.23-2.44, p=0.64) or proximity of 6-month visit completion to due date (hazard ratio 1.07, 95% CI 0.86-1.33, p=0.55). Assessment of assumptions noted that the Kaplan Meier curves crossed, however Grambsh and Therneau testing did not indicate any statistically significant non proportionality (SWAT allocation p=.047; Main trial allocation p=0.52; global test p=0.63).

## Discussion

It is unclear whether an additional £10 in cash increases the likelihood of participants completing a face-to-face follow-up, the proportion of the participants withdrawing, or have an effect on the proximity of the visit to the due date.

### Strengths and limitations

A small positive difference was observed; however, despite the large sample size, the study was underpowered to confidently rule out a small ‘true’ effect. Due to the small effect size (3.1% increase in response) the cost per additional person attending would be in excess of £300.

Due to the sample size of this SWAT, it is most likely generalisable to the larger host trial population of patients with severe mental ill health disorders.

Data was not collected on how study staff followed the guidance on discussing the contingent £10 note to intervention group participants when arranging follow up visits. This may have diluted the effect of the intervention.

## Conclusion

It is unclear if contingent financial incentives increase rates of face-to-face follow-up completion in this trial. However, there were sample size and power limitations. Future SWATs are needed to add to the evidence base.

## Data availability

### Underlying data

Figshare: SCIMITAR+ Trial: A randomised study within a trial (SWAT) of a contingent financial reward to improve trial follow-up - Data Set, https://doi.org/10.6084/m9.figshare.10060202.v2 ^8^.

### Reporting guidelines

Figshare: CONSORT checklist for SCIMITAR+ Trial: A randomised study within a trial (SWAT) of a contingent financial reward to improve trial follow-up, https://doi.org/10.6084/m9.figshare.10060202.v2 ^8^.

Data are available under the terms of the Creative Commons Attribution 4.0 International license (CC-BY 4.0).
