# Supplementary material for: The effectiveness of a contingent financial incentive to improve trial follow up; a randomised study within a trial (SWAT)
Source: F1000Res. 2020 Jul 17;8:1937. Originally published 2019 Nov 20. [Version 2] doi: 10.12688/f1000research.21059.2 (PMC7372525; doi:10.12688/f1000research.21059.2)
Supplement: Peer review history

# Peer review: Reviewer response for version 2

**DOI:** 10.5256/f1000research.27418.r67390

**Published:** 2020-07-20

**Authors:** Frances Shiely

**Affiliations:** 1 School of Public Health, University College Cork, Cork, Ireland; 2 HRB Clinical Research Facility, University College Cork, Cork, Ireland

Thank you to the authors for making these revisions and providing a detailed response to my original queries. I think the revisions have greatly improved the article and am happy to recommend it be accepted.

Is the work clearly and accurately presented and does it cite the current literature?

Partly

If applicable, is the statistical analysis and its interpretation appropriate?

Partly

Are all the source data underlying the results available to ensure full reproducibility?

No source data required

Is the study design appropriate and is the work technically sound?

No

Are the conclusions drawn adequately supported by the results?

Partly

Are sufficient details of methods and analysis provided to allow replication by others?

Yes

Reviewer Expertise:

Epidemiology; trial methodological reserach

I confirm that I have read this submission and believe that I have an appropriate level of expertise to confirm that it is of an acceptable scientific standard.

# Peer review: Reviewer response for version 1

**DOI:** 10.5256/f1000research.23176.r62472

**Published:** 2020-04-24

**Authors:** Sarah A. Rhodes

**Affiliations:** 1 Centre for Biostatistics, School of Health Sciences, University of Manchester, Manchester, UK

1. There seems to be an error in this sentence in the abstract – ‘The difference (3.1%) was not statistically significant (p=0.36; OR 1.29, 95% CI 0.71-2.33, p=0.41).’ – two different p-values are reported and the incorrect one should be removed.
2. This is a paper about the financial incentive SWAT and not the SCIMITAR+ trial – the title seems to suggest that this is the trial report for the SCIMITAR+ trial. Something like ‘The effect of contingent financial reward to improve retention; A randomised study embedded within the SCIMITAR+ trial’ might be better.
3. This SWAT has minimal power to answer a research question by itself, but as the authors rightly say it is an important addition to the body of evidence. I find the term ‘not statistically significant’ potentially misleading because it seems to suggest that the intervention wasn’t effective whereas the confidence intervals are very wide, and appear consistent with previous results. I’d recommend removing reference to ‘statistical significance’ and instead using terms like ‘it is unclear whether….’, given the very wide confidence intervals.
4. The description ‘This SWAT evaluated the effectiveness of a contingent financial reward - £10 cash in addition to a routinely provided £10 voucher - versus no contingent financial reward, on improving the retention rate in the SCIMITAR+ trial’ is difficult to understand. It seems like the intervention is the offer of a contingent financial reward rather than the reward itself (which happens after outcome measurement) – should this perhaps be ‘the effectiveness of the offer of a contingent financial reward’? The £10 voucher happens in both arms so perhaps this should say ‘versus the routinely rewarded £10 voucher alone’ – unless I’m misunderstanding what the control group is here.
5. It says ‘contingent financial reward’ in some places and ‘financial incentive’ in others – are these the same thing? It will be helpful if one term were used consistently.
6. It says that analyses were ‘on an intention to treat basis’ but 11 randomised participants that withdrew before the 6-month visit were excluded from the analysis; this is not really compatible with a true ‘intention to treat’ analysis which aims to preserve randomised groups. Perhaps clearly describe the analysis population rather than calling it ‘intention to treat’ – e.g. the analysis population was participants randomised to the SWAT who had not withdrawn by time X days before due visit. Consider a sensitivity analysis including all randomised participants.
7. The observed effect size was 3.1%; there is lots of uncertainty in this estimate so ideally it shouldn’t really be used to infer the likely cost effectiveness of future studies – instead, I’d recommend considering each end of the confidence interval (or perhaps the upper end as a best-case scenario) when considering cost per additional person retained.
8. I don’t understand the sentence ‘Due to the sample size of this SWAT, it is most likely generalisable to the larger host trial population of patients with severe mental ill health disorders.’ A large sample size does not guarantee generalisability, and as noted the sample size is not really large enough to draw conclusions about this population. I think this point needs to be constructed more clearly.
9. For the secondary outcome of ‘proximity to due date’, I’m really interested to know whether the proportional hazards assumption holds. It would be helpful to include a Kaplain-Meier curve and/or some comment about assumption checking.

Is the work clearly and accurately presented and does it cite the current literature?

Yes

If applicable, is the statistical analysis and its interpretation appropriate?

Partly

Are all the source data underlying the results available to ensure full reproducibility?

Yes

Is the study design appropriate and is the work technically sound?

Yes

Are the conclusions drawn adequately supported by the results?

Partly

Are sufficient details of methods and analysis provided to allow replication by others?

Yes

Reviewer Expertise:

Statistics, randomised trials, embedded trials, meta-analysis.

I confirm that I have read this submission and believe that I have an appropriate level of expertise to confirm that it is of an acceptable scientific standard.

# Response

**Published:** 2020-06-15

**Authors:** Catherine Arundel

**Affiliations:** University of York, UK, UK

There seems to be an error in this sentence in the abstract – ‘The difference (3.1%) was not statistically significant (p=0.36; OR 1.29, 95% CI 0.71-2.33, p=0.41).’ – two different p-values are reported and the incorrect one should be removed.

*We thank the reviewer for this observation. The error has been corrected accordingly*

This is a paper about the financial incentive SWAT and not the SCIMITAR+ trial – the title seems to suggest that this is the trial report for the SCIMITAR+ trial. Something like ‘The effect of contingent financial reward to improve retention; A randomised study embedded within the SCIMITAR+ trial’ might be better.

*We thank the reviewer for this observation. The title has been amended for clarity.*

This SWAT has minimal power to answer a research question by itself, but as the authors rightly say it is an important addition to the body of evidence. I find the term ‘not statistically significant’ potentially misleading because it seems to suggest that the intervention wasn’t effective whereas the confidence intervals are very wide, and appear consistent with previous results. I’d recommend removing reference to ‘statistical significance’ and instead using terms like ‘it is unclear whether….’, given the very wide confidence intervals.

*We agree with the reviewers thoughts here. The manuscript has therefore been updated to remove ‘statistical significance’ and to make clear the lack of certainty. P values have however been retained for completeness.*

The description ‘This SWAT evaluated the effectiveness of a contingent financial reward - £10 cash in addition to a routinely provided £10 voucher - versus no contingent financial reward, on improving the retention rate in the SCIMITAR+ trial’ is difficult to understand. It seems like the intervention is the offer of a contingent financial reward rather than the reward itself (which happens after outcome measurement) – should this perhaps be ‘the effectiveness of the offer of a contingent financial reward’? The £10 voucher happens in both arms so perhaps this should say ‘versus the routinely rewarded £10 voucher alone’ – unless I’m misunderstanding what the control group is here.

*We have amended the manuscript in line with the reviewer’s suggestion to make clearer the control group.*

It says ‘contingent financial reward’ in some places and ‘financial incentive’ in others – are these the same thing? It will be helpful if one term were used consistently.

*We apologise for this inconsistency. The manuscript has been updated throughout to ensure consistency,*

It says that analyses were ‘on an intention to treat basis’ but 11 randomised participants that withdrew before the 6-month visit were excluded from the analysis; this is not really compatible with a true ‘intention to treat’ analysis which aims to preserve randomised groups. Perhaps clearly describe the analysis population rather than calling it ‘intention to treat’ – e.g. the analysis population was participants randomised to the SWAT who had not withdrawn by time X days before due visit. Consider a sensitivity analysis including all randomised participants.

*We can see why the reviewer feels that this is not strictly ITT. The study randomisation was done in a single batch, rather than when the participant reached the Month 6 time point, and so participants received an allocation when they were not technically eligible for the SWAT, hence the exclusion.*

**

*As a result, we suggest that the analysis in the paper is ITT in the sense that the analysis includes everyone who would have been eligible for the SWAT. We have sought to make this clearer in the paper for avoidance of confusion.*

**

*For added confidence, the analysis has been re-run including those who withdrew post randomisation, but pre visit. When these are included the findings do not change **.***

**

The observed effect size was 3.1%; there is lots of uncertainty in this estimate so ideally it shouldn’t really be used to infer the likely cost effectiveness of future studies – instead, I’d recommend considering each end of the confidence interval (or perhaps the upper end as a best-case scenario) when considering cost per additional person retained.

*We thank the reviewer for making this important point.*

*As the manuscript states, the cost of retaining an additional participant would be over £300. This statement is still correct in the worst case scenario, and as such covers the uncertainty; whilst also covering the estimate that we have found in this study.*

**

I don’t understand the sentence ‘Due to the sample size of this SWAT, it is most likely generalisable to the larger host trial population of patients with severe mental ill health disorders.’ A large sample size does not guarantee generalisability, and as noted the sample size is not really large enough to draw conclusions about this population. I think this point needs to be constructed more clearly.

*For clarity, this section has been amended to reflect that results are only applicable to the study population.*

For the secondary outcome of ‘proximity to due date’, I’m really interested to know whether the proportional hazards assumption holds. It would be helpful to include a Kaplain-Meier curve and/or some comment about assumption checking.

*The results section has been updated to detail assessment of hazards assumptions.*

# Peer review: Reviewer response for version 1

**DOI:** 10.5256/f1000research.23176.r56823

**Published:** 2019-12-03

**Authors:** Frances Shiely

**Affiliations:** 1 School of Public Health, University College Cork, Cork, Ireland; 2 HRB Clinical Research Facility, University College Cork, Cork, Ireland

Thank you for the opportunity to review this SWAT. I am in favour of publishing negative results as well as positive results, so my reason for rejecting this SWAT is not because the findings are negative. I wish to be clear on that. 

 This was a SWAT within the SCIMITAR+ trial. The purpose of the SWAT was to establish if £10 cash + £10 voucher would increase retention rates when compared to £10 voucher only. SCIMITAR+ was a smoking cessation trial. The authors conclude that contingent financial incentives did not statistically significantly increase rates of face-to-face follow-up completion within the SCIMITAR+ trial.

 In the background section of the abstract, you describe the SWAT as  “…versus no contingent financial incentive” which is misleading. There is a contingent financial incentive because you have a £10 voucher as standard. I presume it’s contingent on them turning up to the appointment? I had to go to the body of the paper to ensure I had the correct interpretation. You should describe the SWAT more accurately as “to establish if £10 cash + £10 voucher would increase retention rates when compared to £10 voucher only” and change this in the main body of the paper also.

 I struggled with the design of this SWAT and what the investigators hoped to achieve by it. I don’t think the primary outcome was suitable to measure what they intended. The conclusion drawn in the abstract is unrelated to the primary outcome. The purpose of the SWAT was to look at retention. The participants were eligible for the SWAT if they reached the SCIMITAR+ 6-month follow-up. I’d like the authors to comment on this in relation to their SWAT. It could be argued that if they were already coming for the 6 month follow-up they were motivated to continue regardless of the extra financial incentive. It seems like 100% of those contacted for the 6-month follow-up turned up to their appointment. Therefore, 100% were retained. I don’t see the point then in making the primary outcome contingent on the CO breath measurement and was not surprised there was no statistically significant difference between the two groups. Your secondary outcome, the proportion withdrawing after the contact would be more relevant. This is supported by the fact you used the proportion turning up for the appointment as your concluding statement.

 Comparing two incentivised arms at 6 month follow-up doesn’t appear to be the best use of the money. What would have been interesting to find out is if the additional money at 12 months would make a difference to retention? Perhaps let the intervention arm know at 6-months that at 12 months, in addition to the voucher they would get £10 cash. Then you could establish if the additional incentive was effective. 

 I would have liked to know what you told the comparator group when you phoned them to come to their 6-month visit. Did you tell them they were getting a £10 voucher to give a CO. Did you make them aware of what the intervention group were getting or did you tell them about the SWAT at all?

 The participants were already incentivised with the £10 voucher. What is the evidence base for thinking that an additional £10 cash would increase retention at 6 months? I note the Bailey et al. 2013 study but this was in relation to response rates for a postal questionnaire. ^1^

Is the work clearly and accurately presented and does it cite the current literature?

Partly

If applicable, is the statistical analysis and its interpretation appropriate?

Partly

Are all the source data underlying the results available to ensure full reproducibility?

No source data required

Is the study design appropriate and is the work technically sound?

No

Are the conclusions drawn adequately supported by the results?

Partly

Are sufficient details of methods and analysis provided to allow replication by others?

Yes

Reviewer Expertise:

Epidemiology; trial methodological reserach

I confirm that I have read this submission and believe that I have an appropriate level of expertise to state that I do not consider it to be of an acceptable scientific standard, for reasons outlined above.

## References

[1] : The Sexunzipped trial: optimizing the design of online randomized controlled trials. *J Med Internet Res*.2013;15(12) : 10.2196/jmir.2668 e278 10.2196/jmir.2668 24334216PMC3868980

# Response

**Published:** 2020-06-15

**Authors:** Catherine Arundel

**Affiliations:** University of York, UK, UK

In the background section of the abstract, you describe the SWAT as  “…versus no contingent financial incentive” which is misleading. There is a contingent financial incentive because you have a £10 voucher as standard. I presume it’s contingent on them turning up to the appointment? I had to go to the body of the paper to ensure I had the correct interpretation. You should describe the SWAT more accurately as “to establish if £10 cash + £10 voucher would increase retention rates when compared to £10 voucher only” and change this in the main body of the paper also.

*We thank the reviewer for this helpful suggestion. We have amended the manuscript to make clearer the control group.*

 I struggled with the design of this SWAT and what the investigators hoped to achieve by it. I don’t think the primary outcome was suitable to measure what they intended. The conclusion drawn in the abstract is unrelated to the primary outcome.

 The purpose of the SWAT was to look at retention. The participants were eligible for the SWAT if they reached the SCIMITAR+ 6-month follow-up. I’d like the authors to comment on this in relation to their SWAT. It could be argued that if they were already coming for the 6 month follow-up they were motivated to continue regardless of the extra financial incentive.

   

 It seems like 100% of those contacted for the 6-month follow-up turned up to their appointment. Therefore, 100% were retained. I don’t see the point then in making the primary outcome contingent on the CO breath measurement and was not surprised there was no statistically significant difference between the two groups. Your secondary outcome, the proportion withdrawing after the contact would be more relevant. This is supported by the fact you used the proportion turning up for the appointment as your concluding statement.

*We thank the reviewer for their comments here and apologise for any confusion.*

*Having reviewed, we believe that the conclusion does relate to the primary outcome given it was unclear, due to the wide confidence intervals if there was any difference in completion of a CO breath measure between the two groups.*

*Following review of this, and comments from Reviewer 2, we can see there has been some misunderstanding. The study randomisation was done in a single batch, rather than when the participant reached the Month 6 time point, therefore at the time of randomisation it was no known if the participant would attend their 6 month visit. We have made this clearer in the randomisation and results sections.*

*We are clear how the reviewer has derived this information. Figure 1 demonstrates that 32 intervention and 21 control participants did not attend a face to face visit and so did not provide a CO breath measure.*

 Comparing two incentivised arms at 6 month follow-up doesn’t appear to be the best use of the money. What would have been interesting to find out is if the additional money at 12 months would make a difference to retention? Perhaps let the intervention arm know at 6-months that at 12 months, in addition to the voucher they would get £10 cash. Then you could establish if the additional incentive was effective. 

*The impact of the contingent reward on subsequent follow up time points is an important consideration. The effects were not assessed in this study, but this would be important to include in any subsequent studies of this nature.*

 I would have liked to know what you told the comparator group when you phoned them to come to their 6-month visit. Did you tell them they were getting a £10 voucher to give a CO. Did you make them aware of what the intervention group were getting or did you tell them about the SWAT at all?

*As noted in the intervention description, during the call to arrange a follow up appointment intervention participants were advised of the contingent £10 if they were willing to meet face to face and provide the primary outcome. The intervention section has been reviewed to make this clearer.*

 The participants were already incentivised with the £10 voucher. What is the evidence base for thinking that an additional £10 cash would increase retention at 6 months? I note the Bailey et al. 2013 study but this was in relation to response rates for a postal questionnaire. ^1^

*There is much available evidence on strategies to improve postal questionnaire response rates, but limited evidence for strategies to improve face to face visit completion.*

*We suggest that strategies used for postal questionnaire response rates may have similar effectiveness for face to face visits, hence the completion of this SWAT. We have made clear in the background the justification for this.*
